# Unraveling the intersection of sleep disorders and erectile dysfunction: Outcomes from two EPISONO editions

**DOI:** 10.1111/andr.70067

**Published:** 2025-06-04

**Authors:** Monica Levy Andersen, Allan Saj Porcacchia, Guilherme Luiz Fernandes, Tathiana A. Alvarenga, Sergio Tufik

**Affiliations:** ^1^ Departamento de Psicobiologia Universidade Federal de São Paulo (UNIFESP) São Paulo Brazil; ^2^ Instituto do Sono São Paulo Brazil

**Keywords:** apnea‒hypopnea index, epidemiology, erectile dysfunction, incidence, prevalence, psychological well‐being, sleep, sleep apnea, testosterone

## Abstract

**Background:**

There is growing interest in the relationship between sleep disorders and erectile dysfunction. We present the results from a 2015 follow‐up study in relation to the 2007 edition of Epidemiologic Sleep Study (EPISONO), a population‐based sleep study conducted in São Paulo, Brazil, and from the 4th edition of EPISONO (2018), with respect to the incidence and prevalence of erectile dysfunction, and the associated risk factors. We hypothesized that the presence of erectile dysfunction would be associated with total testosterone levels and obstructive sleep apnea in both longitudinal and cross‐sectional analyses.

**Method:**

The participants underwent polysomnography, and testosterone assays were performed. They also completed a range of health questionnaires. The sample comprised men aged 20‒80 years. In the longitudinal analysis, incidence the (*N* = 256) and prevalence (*N* = 300) of erectile dysfunction were calculated, and a generalized estimating equation model was constructed. For the analysis of the data from 2018, prevalence (*N* = 314), binomial logistic regression, and mediation‐moderation models were calculated.

**Results:**

An overall incidence of 10.55% of erectile dysfunction was found in the follow‐up, which was higher in men older than 50 years. In the longitudinal model, older age (odds ratio = 1.09), more depression symptoms (odds ratio = 1.05), and low total testosterone concentration (odds ratio = 2.69) were significant predictors of erectile dysfunction. Psychological well‐being (World Health Organization Quality of Life) was a predictor of lowered odds of having erectile dysfunction (odds ratio = 0.87). In EPISONO 2018, a 20.06% general prevalence of erectile dysfunction was identified, and this prevalence was higher in age groups over 50 years. The odds of having erectile dysfunction were increased by age (odds ratio = 1.07), and the psychological domain of World Health Organization Quality of Life was associated with lowered odds of having erectile dysfunction (odds ratio = 0.65). Mediation models revealed a statistically significant mediation of apnea‒hypopnea index between the effect of age on total testosterone. The model that included age as the independent variable, apnea‒hypopnea index as a mediator and erectile dysfunction as the outcome resulted in significant effects of age but not apnea‒hypopnea index.

**Conclusions:**

This study highlights the importance of aging, psychological quality of life, testosterone concentration, and depressive symptoms in the context of erectile dysfunction. An association between obstructive sleep apnea and erectile dysfunction was observed, but it was not independent of age. The longitudinal results emphasize that, besides aging, these are modifiable factors that can be the subject of interventions to mitigate the development of erectile dysfunction over time.

## 1 INTRODUCTION

Erectile dysfunction (ED) is characterized by the persistent or recurring inability to achieve or maintain an erection sufficient for satisfactory sexual intercourse.^1^ Often referred to as impotence, ED can have a significant impact on the well‐being of those with the condition.^2^ Its effects extend beyond physical limitations, influencing emotional and psychological aspects, and potentially leading to distress, anxiety, and interpersonal difficulties.^3^ ED has diverse underlying causes, encompassing various health conditions, lifestyle choices, and psychological factors, including depression.^3^ Decreased androgen levels are another known factor related to ED, emphasizing the importance of hormonal evaluation in patients with ED. Thus, it is crucial to acknowledge the broad range of factors that can contribute to ED. Recently, researchers have increasingly been exploring the relationship between sleep disorders and ED, recognizing sleep as a vital physiological process crucial for overall health.^4^ There is growing evidence that sleep disorders—especially obstructive sleep apnea (OSA)^5^, ^6^, ^7^—can disrupt this essential function and significantly impact individuals' daily lives.^8^

Our group published a study in 2010, using data from the 2007 edition of the ongoing Epidemiologic Sleep Study (EPISONO; a population‐based sleep study being carried out in São Paulo, Brazil), to estimate the prevalence of OSA. The sample comprised 1042 participants who underwent polysomnography (PSG), completed a series of questionnaires and provided fasting blood samples to collect data on a range of sleep parameters and health conditions. It was found that OSA was present in 32.89% of the participants.^9^ In this EPISONO study, which was based on the same data, the overall prevalence of ED complaints was 17.08%, ranging from 7.3% in younger men (20‒29 years old) to 63.25% in older men (>60 years old).^6^ Reduced time spent in rapid eye movement (REM) sleep, fragmented sleep, obesity, low testosterone levels, low quality of life, an apnea‒hypopnea index (AHI) of over 15 events/h, and OSA were significantly associated with a higher risk of ED complaints.^6^ The study concluded that ED complaints were relatively common, particularly among older men, and that adequate sleep patterns and normal or high levels of testosterone may be protective against ED, whereas an AHI over 15 events/h increased the odds of having ED, and subsequently affected sexual activity.^6^ However, it was still necessary to assess whether these associations would be maintained over time.

After the 2007 edition of EPISONO, another two new surveys were completed: a follow‐up study was conducted in 2015 with the aim of assessing the progression of sleep complaints and the development of comorbidities and ED in the same group of volunteers from the 2007 study, and the 4th edition of EPISONO was conducted in 2018, with a new population sample. Here, we use data from the follow‐up study, and the 4th edition of EPISONO. The study hypothesis was that sleep disorders, namely, OSA, as measured by one of its main markers, AHI, along with low testosterone, depression symptoms, and low score in the psychological domain of World Health Organization Quality of Life (WHOQOL) would be significant predictors of ED in a longitudinal setting and significantly associated with ED in the 2018 dataset.

## 2 METHODS

### 2.1 Population

EPISONO is a population‐based study of sleep disturbances and their risk factors in the population of São Paulo, the largest city in Brazil. São Paulo is home to 11,452 million residents, with an area of 1521 km^2^ and a population density of 7528 inhabitants/km^2^, which varies considerably across different districts (https://cidades.ibge.gov.br/brasil/sp/sao‐paulo/panorama). It exhibits significant ethnic diversity, stemming from its history of slavery with the arrival of African slaves in the 16th to 19th centuries, the presence of indigenous populations, as well as waves of migration from Europe and Asia in the 19th and 20th centuries.

### 2.2 Sampling

The sampling procedure was carried out to represent the adult population of the city of São Paulo in terms of sex, age, and socioeconomic status in both 2007^10^ and 2018. The sample consists of men and women, aged between 20 and 80 years, belonging to low, middle, and high socioeconomic statuses, in proportion to the population distribution.

Representative samples were determined by stratified sampling at four levels (districts, blocks, households, and individuals). Twelve municipal districts were randomly selected from four homogeneous regions representing the economically active population of São Paulo, according to past surveys (1987, 1995, and 2007). From these areas, census tracts, smaller areas used to gather census information (Instituto Brasileiro de Geografia e Estatística), with approximately 200 households each, were sampled, with an average of 25 households per tract. In the chosen sector, permanently occupied private residences were selected. Areas at risk or potentially dangerous, such as slums and tenements, were excluded to prioritize the safety of interviewers. The selection of individual households was made by the random selection of the first number and then skipping the interval of households relative to the total number of households per sector divided by 25, following a specific order. Regarding apartment buildings, each apartment was considered a household, and counting occurred from the top floor downwards. The selection of the individual to be interviewed per household was established by random criteria. The sample size calculation was based on data obtained from the previous edition of EPISONO undertaken in 2007 (1042 participants; 468 men; the number of male participants was recounted and updated because of the 2010 related publication) and updated population data for the city of São Paulo, according to estimates based on the 2010 Demographic Census. In the 2015 follow‐up, there were 688 participants (308 men), and in the 2018 sample, there were 769 individuals (317 men).

### 2.3 Design

In both studies, the participants completed two research stages, the first being undertaken in the household and the second at the Sleep Institute. At the household stage, interviewers visited the randomized volunteers' residences. After agreeing to take part in the study, reading and signing an informed consent form, a household survey questionnaire that gathered personal information and socioeconomic data was administered, and the date of the visit to the Sleep Institute/Associação Fundo de Incentivo à Pesquisa was scheduled.

The visit to the Sleep Institute took place in the same week as the household visit. Volunteers underwent anthropometric evaluation (measurement of weight, height, neck, hip, and waist circumferences), completed the questionnaires, and underwent full‐night PSG, which respected the volunteers’ habitual sleep times. The participants received dinner before institutional data collection. The following morning, blood collection was performed for biochemical assays.

**FIGURE 1:**
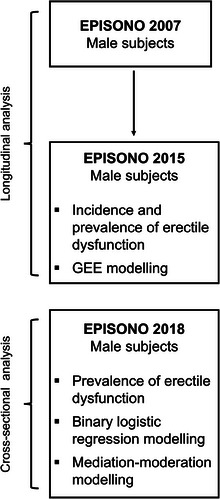
Flowchart summarizing the design of the analysis presented in the study.

The current study presents two analyses with different designs: a longitudinal analysis with data from 2015, which was a follow‐up of the 2007 study,^6^ and a cross‐sectional analysis with data from a new sample collected in 2018, as shown in Figure 1. Both studies have been previously approved by the UNIFESP Ethics Committee (CEP 610.516 and 2.252.970, respectively).

### 2.4 Inclusion and exclusion criteria

The inclusion criteria for both analyses were male individuals who completed the questionnaire on sexual performance/ED. For the incidence and prevalence rate calculations, subjects who did not respond to the question “How would you describe your ability to get and keep an erection that is adequate for satisfactory intercourse?” in 2007, 2015, and 2018 were excluded. For the statistical modeling analysis, missing data for any of the included variables were considered an exclusion criterion.

### 2.5 Questionnaires

The questionnaires completed by the participants at the Sleep Institute and used in this study were the Questionnaire of Sexual Performance/ED for Men,^11^, ^12^ the Beck Depression Inventory (BDI),^13^, ^14^ the Beck Anxiety Inventory (BAI),^13^, ^15^ and the WHOQOL instrument.^16^, ^17^ All the participants were asked to declare whether they had/have diabetes or stroke history.

### 2.6 Polysomnography

The full‐night PSG was conducted using a digital EMBLA system (N7000, Embla Systems Inc.) following the current recommendations of the American Academy of Sleep Medicine (AASM). All recording sensors were non‐invasively affixed to the individual using adhesive tape and/or elastic collodion. The following physiological variables were simultaneously and continuously monitored: six channels of electroencephalogram (F3‒A2, F4‒A1, C3‒A2, C4‒A1, O1‒A2, and O2‒A1); two channels of electrooculogram (EOG) (E1‒M2 and E2‒M2); three channels of chin electromyogram (EMG); and one channel of the masseter muscle EMG, one channel of the temporal muscle EMG, and one channel of the electrocardiogram (modified D2 derivation). Airflow was detected in two channels using a thermocouple and an EMBLA pressure transducer (for nasal airflow). For thoracic and abdominal respiratory effort, two channels were used for X‐trace belts (EMBLA). Another two channels were used for snore sensors and body position (EMBLA). Oxygen saturation (SaO_2_) assessment was performed using an EMBLA pulse oximeter.

Sleep staging was visually performed by four technicians trained by an AASM‐certified technologist, according to the criteria of the AASM 2007^18^ manual and the criteria of the new 2012 AASM manual.^19^ Awakenings and periodic leg movements were analyzed according to the AASM 2007^18^ manual and 2012 manual.^19^ Apneas and hypopneas were scored according to the recommended rule in the 2012 AASM manual.^19^ Technicians were blinded to each analysis, and there was a reader agreement analysis for 10% of the randomly selected examinations.

### 2.7 Definition of erectile dysfunction

The presence of ED complaints was determined through the question “How would you describe your ability to get and keep an erection that is adequate for satisfactory intercourse?” in the Questionnaire of Sexual Performance/ED for Men with four possible answers, always/usually/sometimes/never. The answers “sometimes” or “never” were considered as presence of ED. This question was designed based on the recommendation of the National Institutes of Health Consensus Development Panel on Impotence^11^ and has shown appropriate accuracy in identifying ED.^12^

### 2.8 Biochemical and hematological assays

Blood sample collection was performed for serum and plasma assays. Total testosterone assays were performed using the chemiluminescence method.

### 2.9 Statistical analysis

The general and age‐group ED prevalence was calculated for data from 2015 and 2018. The general ED incidence and incidence by age group (considering age in 2015) were calculated according to the formula below. Only participants with ED data at both timepoints were included in the incidence rate calculation.

$$\frac{\mathrm{New}\;\mathrm{cases}\;\mathrm{of}\;\mathrm{ED}\;\mathrm{in}\;2015}{\mathrm{Cases}\;\mathrm{without}\;\mathrm{ED}\;\mathrm{in}\;2007}$$

A generalized estimating equations (GEE) model was used to evaluate the effects of the independent variables—AHI, WHOQOL psychological domain score, BDI score, and total testosterone (threshold of <300 ng/dL for low testosterone)^20^—on the odds of having ED. The model had a binary logistic probability distribution and was controlled for age and body mass index (BMI) in 2007. The covariance matrix was set as autoregressive 1.

For the cross‐sectional study, a binary logistic regression was performed including the following independent variables: age, total testosterone, WHOQOL psychological domain score, AHI (<15 or ≥15),^21^ arterial hypertension (no or yes), diabetes (no or yes), race/skin color (white, black, mixed race, or indigenous/Asian/other/unknown, merged because of the low frequency of these race/skin colors in the sample), BMI (normal, overweight, or obese),^22^ BDI score, and BAI score.

Descriptive statistics were conducted for both study designs and Jamovi (version 2.4) was used to run GEE and binary logistic regression models, considering a statistical significance level of *p* < 0.05. Sample weights were not employed in any analysis.

The mediation/moderation modeling was conducted with data from 2018 to evaluate the relationship between age, total testosterone, AHI, and ED. Four models were estimated: (i) the direct effect of age on ED, considering AHI as the indirect effect [*a* + (*b* × *c*)]; (ii) the direct effect of age on total testosterone, considering AHI as the mediator [*d* + (*e* × *f*)]; (iii) the direct effect of AHI on ED, considering total testosterone as the indirect effect [*g* + (*h* × *i*)]; (iv) the direct effect of age on ED, with total testosterone as a mediator [*j* + (*k* × *l*)]. This analysis was performed in R using the *lavaan* package, the estimator was diagonally weighted least squares for models i, iii, and iv because of the binary outcome ED and maximum likelihood for model ii; the effects were statistically significant when *p* < 0.05.

## 3 RESULTS

### 3.1 Longitudinal analysis (EPISONO 2007 and follow‐up in 2015)

**FIGURE 2:**
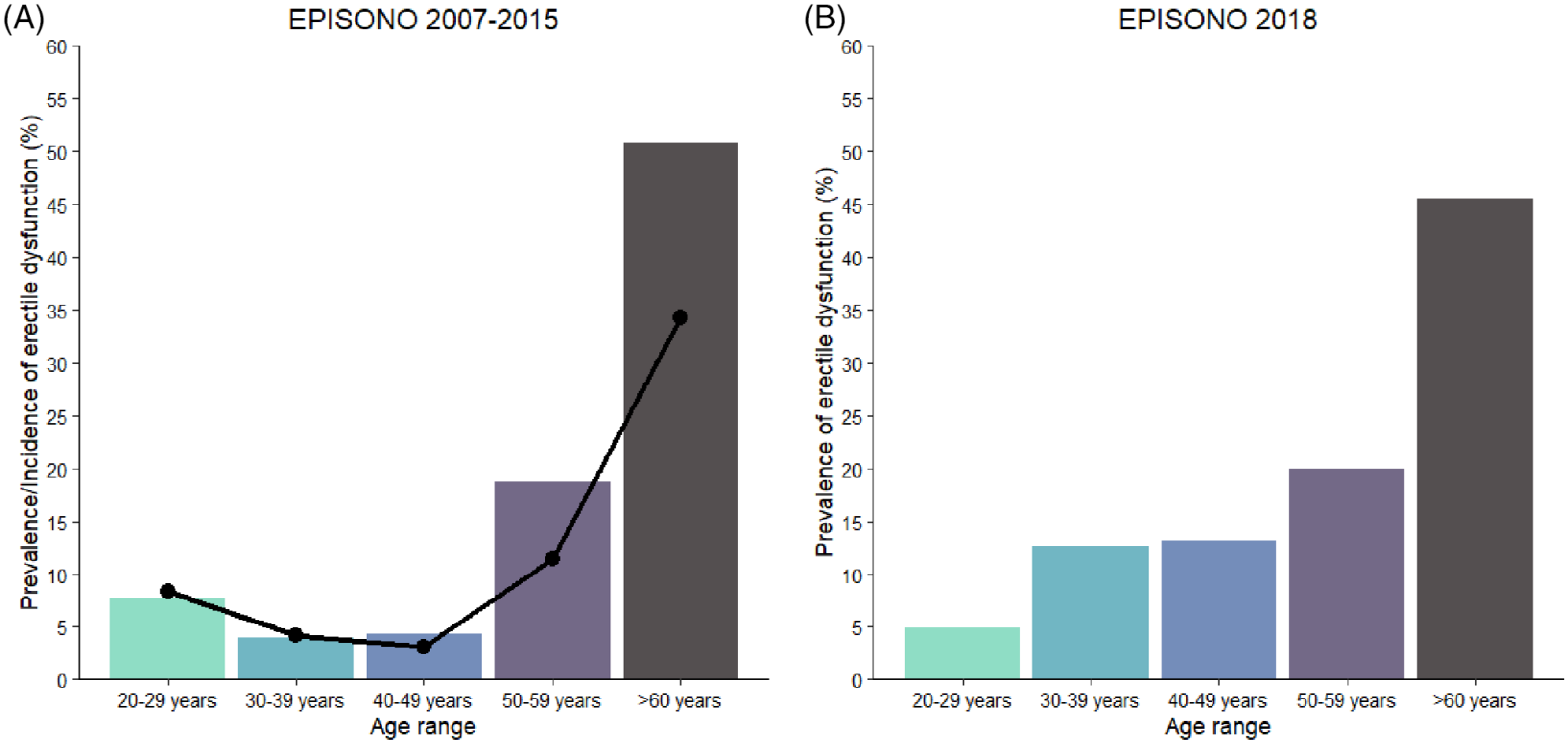
Bar plots representing the prevalence of erectile dysfunction (ED) by age group. (A) Bars show the prevalence ED in 2015, and the curve represents the 8‐year incidence of this dysfunction according to age groups, increasing sharply after 50 years of age. (B) Correspondence to the prevalence of ED in 2018.

**FIGURE 3:**
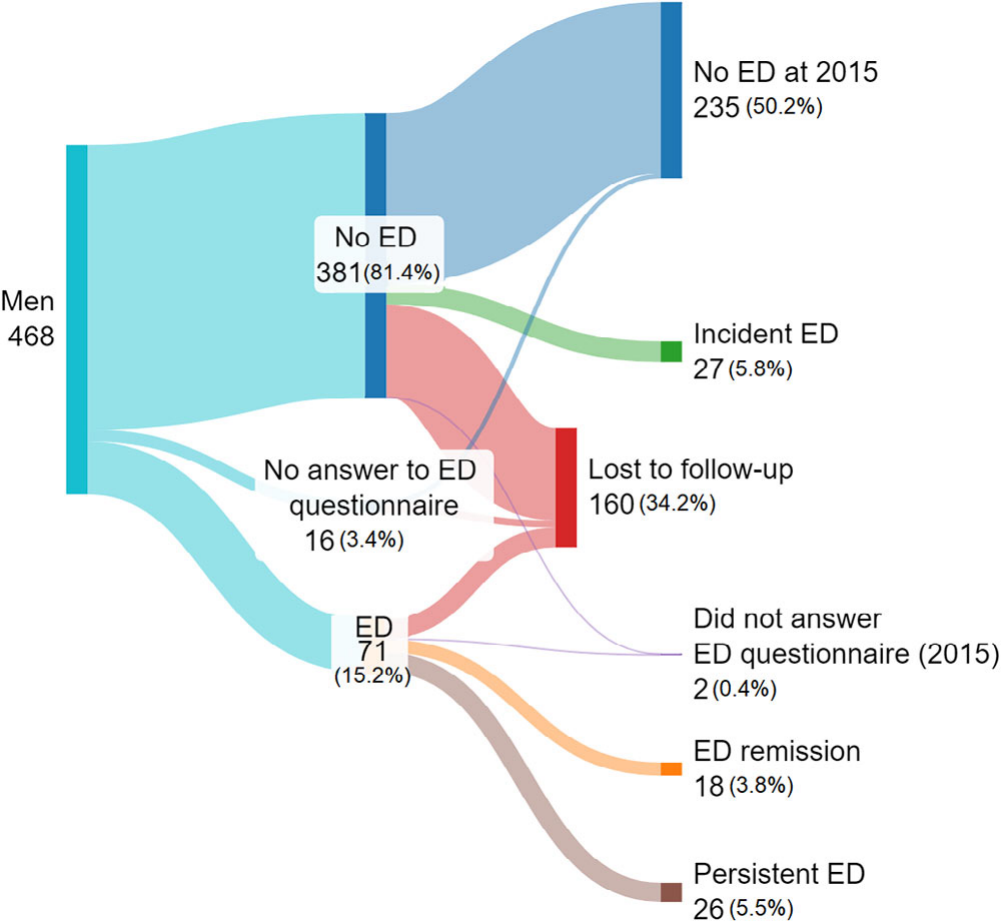
Sankey diagram showing the epidemiological trajectory of erectile dysfunction (ED) in the 2007 and 2015 EPISONO data collections. In this diagram, relative frequencies (%) represent the proportion of the category among total answers in each year.

The overall incidence of ED was 10.55% (95% confidence interval [CI] = [7.2%; 14.7%]), with 27 new cases, and 235 subjects did not have ED in 2015. The incidence rates of age groups 50–59 years and over 60 years were 11.43% and 34.21%, respectively. The general prevalence of ED in 2015 was 17.67% (95% CI = [13.6%; 22.2]) and the age groups 50–59 years and over 60 years presented higher values, being 18.75% and 50.82%, respectively (Figure 2A and Table 1). The changes in ED (Figure 3) were that from the 71 participants with ED in the first survey in 2007, 18 had remission in 2015, while 26 persisted with ED. There was a 34.61% loss of subjects in the follow‐up, and two participants did not answer the ED questionnaire in the follow‐up study.

**TABLE 1:** Incidence of erectile dysfunction (ED) (only respondents of EPISONO 2007 and its follow‐up in 2015) and prevalence of ED in 2015 and 2018.

	Incidence of ED in 2007‒2015 (*N* = 256^a^)	95% CI	Prevalence of ED in 2015 (*N* = 300)	95% CI	Prevalence of ED in 2018 (*N* = 314)	95% CI
General sample	27/256 (10.55%)	[7.2%; 14.7%]	53/300 (17.67%)	[13.6%; 22.2%]	63/314 (20.06%)	[15.9%; 24.7%]
20‒29 years	1/12 (8.33%)		1/13 (7.69%)		2/41 (4.88%)	
30‒39 years	3/72 (4.17%)		3/76 (3.95%)		9/71 (12.68%)	
40‒49 years	2/64 (3.13%)		3/70 (4.29%)		10/76 (13.16%)	
50‒59 years	8/70 (11.43%)		15/80 (18.75%)		12/60 (20.00%)	
≥60 years	13/38 (34.21%)		31/61 (50.82%)		30/66 (45.45%)	

Abbreviation: CI, confidence interval.

^a^ The total sample size in this calculation represents participants with ED data in both timepoints and did not present ED in 2007.

In the GEE model (Table 2), participants who had missing data in any of the variables were excluded; thus, in total, 443 participants were included. The descriptive data of the groups with and without ED are shown in Table 3. BMI and the presence of OSA did not have an association with ED (*p*‐values = 0.926 and 0.728, respectively). Age had a significant association in the model, presenting an odds ratio of 1.091 (95% CI = [1.069; 1.114]; *p* < 0.001). The BDI score had statistical significance, with an odds ratio of 1.051 (95% CI = [1.015; 1.088]; *p* = 0.005). Regarding total testosterone, those who had a low concentration (<300 ng/dL) had an odds ratio of 2.687 for having ED (95% CI = [1.509; 4.784]; *p* = 0.001) compared with those with normal levels. The follow‐up study (odds ratio [OR] = 0.514; 95% CI = [0.322; 0.82]; p = 0.005) and the psychological domain score of WHOQOL (OR = 0.867; 95% CI = [0.761; 0.987]; p = 0.032) had significant negative associations with ED. In summary, having low total testosterone, increasing age and higher scores on BDI increased the odds of having ED over 8 years of follow‐up. On the other hand, a greater psychological domain score and the follow‐up study lowered the odds of having ED.

**TABLE 2:** Results from the generalized estimating equation modeling considering the occurrence (no or yes) of erectile dysfunction as the dependent variable and using longitudinal data from 2008 and the 2015 follow‐up (*N* = 443).

Variables	Estimates	SE	*p*‐value	OR	95% CI
Intercept	‒4.241	1.486	0.004	0.014	[0.001; 0.265]
Age (years)	0.087	0.010	<0.001	1.091	[1.069; 1.114]
BMI	0.002	0.026	0.926	1.002	[0.953; 1.055]
WHOQOL psychological (%)	‒1.430	0.066	0.032	0.867	[0.761; 0.987]
BDI score	0.049	0.017	0.005	1.051	[1.015; 1.088]
Time (2015–2007)	‒0.666	0.238	0.005	0.514	[0.322; 0.820]
AHI (≥15 to <15 events/h)	0.087	0.249	0.728	1.090	[0.669; 1.777]
Total testosterone (<300 to ≥300 ng/dL)	0.988	0.294	0.001	2.687	[1.509; 4.784]

*Note*: ORs resulted from the comparison of having erectile dysfunction—not having erectile dysfunction.

Abbreviations: AHI, apnea‒hypopnea index; BDI, Beck Depression Inventory; BMI, body mass index; CI, confidence interval; OR, odds ratio; SE, standard error; WHOQOL, World Health Organization Quality of Life.

**TABLE 3:** Characteristics of the sample considered for the generalized estimating equation analysis using data from the EPISONO 2007 and follow‐up in 2015.

	2007				2015			
	Without ED (*N* = 381)		With ED (*N* = 71)		Without ED (*N* = 253)		With ED (*N* = 53)	
	Mean (SD)	95% CI	Mean (SD)	95% CI	Mean (SD)	95% CI	Mean (SD)	95% CI
Age (years)	38.349 (12.439)	[37.096; 39.602]	53.155 (15.859)	[49.401; 56.909]	45.929 (11.298)	[44.530; 47.328]	61.811 (12.862)	[58.266; 65.356]
BMI	26.448 (4.698)	[25.974; 26.922]	27.609 (5.640)	[26.264; 28.954]	27.481 (5.329)	[26.821; 28.140]	27.735 (4.840)	[26.387; 29.082]
AHI (events/h)	9.218 (12.684)	[7.940; 10.495]	16.777 (19.308)	[12.207; 21.348]	18.728 (18.331)	[16.459; 20.998]	29.985 (24.905)	[23.120; 36.850]
WHOQOL psychological (%)	15.301 (1.874)	[15.104; 15.498]	14.339 (1.900)	[13.865; 14.814]	15.510 (1.895)	[15.280; 15.740]	15.019 (2.151)	[14.426; 15.612]
BDI score	7.403 (6.716)	[6.699; 8.107]	10.531 (6.441)	[8.922; 12.140]	6.877 (7.251)	[5.980; 7.775]	10.113 (8.781)	[7.693; 12.533]

	*N* (%)		*N* (%)		*N* (%)		*N* (%)	
Total testosterone (ng/dL)								
<300	14 (3.80)		11 (15.90)		53 (20.90)		21 (39.60)	
≥300	356 (96.20)		58 (84.10)		200 (79.10)		32 (60.40)	
AHI (events/h)								
≥15	79 (20.70)		42 (59.20)		114 (45.10)		19 (35.80)	
<15	302 (79.30)		29 (40.80)		139 (54.90)		34 (64.20)	

Abbreviations: AHI, apnea‒hypopnea index; BDI, Beck Depression Inventory; BMI, body mass index; CI, confidence interval; ED, erectile dysfunction; SD, standard deviation; WHOQOL, World Health Organization Quality of Life.

### 3.2 Cross‐sectional analysis (EPISONO 2018)

#### 3.2.1 Prevalence and binary logistic regression model

According to the EPISONO 2018 edition, a 20.06% (95% CI = [15.9%; 24.7%]) general prevalence of ED was identified in the male participants (*N* = 314). The prevalence rate was higher in the age group over 60 years old (45.45%), while the age groups 20–29 years, 30–39 years, and 40–49 years old had prevalence rates of 4.88%, 12.68%, and 13.16%, respectively (Figure 2B and Table 1). The descriptive data of the sample are depicted in Table 4.

**TABLE 4:** Baseline characteristics of the sample from the EPISONO 2018 employed in the binomial logistic regression.

	Total (*N* = 314)		Without ED (*N* = 251)		With ED (*N* = 63)	
	Mean (SD)	95% CI	Mean (SD)	95% CI	Mean (SD)	95% CI
Age (years)	46.465 (14.524)	[44.852; 48.078]	44.104 (13.539)	[42.42; 45.787]	55.873 (14.605)	[52.195; 59.551]
WHOQOL psychological (%)	13.872 (1.830)	[13.666; 14.079]	14.167 (1.549)	[13.971; 14.363]	12.699 (2.339)	[12.100; 13.299]
BDI score	8.049 (7.586)	[7.193; 8.906]	7.259 (6.497)	[6.438; 8.080]	11.197 (10.382)	[8.538; 13.856]
BAI score	6.378 (7.595)	[5.521; 7.235]	6.198 (7.396)	[5.263; 7.132]	7.098 (8.368)	[4.955; 9.242]

	Frequency (%)		Frequency (%)		Frequency (%)	
Total testosterone (ng/dL)						
<300	271 (87.1)		217 (87.15)		54 (87.10)	
≥300	40 (12.9)		32 (12.85)		8 (12.90)	
AHI (events/h)						
<15	227 (72.3)		192 (76.49)		35 (55.56)	
≥15	87 (27.7)		59 (23.51)		28 (44.44)	
Arterial hypertension						
No	229 (75.3)		190 (78.19)		39 (63.90)	
Yes	75 (24.7)		53 (21.81)		22 (36.10)	
Diabetes						
No	271 (89.1)		222 (91.36)		49 (80.33)	
Yes	33 (10.9)		21 (8.64)		12 (19.67)	
Ethnicity			
White	172 (56.8)		146 (60.08)		26 (43.33)	
Black	40 (13.2)		30 (12.35)		10 (16.67)	
Mixed race	56 (18.5)		41 (16.87)		15 (25.00)	
Indigenous/Asian/others/unknown	35 (11.6)		26 (10.70)		9 (15.00)	
BMI						
Normal	101 (32.5)		85 (33.73)		16 (25.40)	
Overweight	128 (41.2)		100 (39.68)		28 (44.44)	
Obese	82 (26.4)		63 (26.59)		19 (30.16)	

Abbreviations: AHI, apnea‒hypopnea index; BAI, Beck Anxiety Index; BDI, Beck Depression Inventory; BMI, body mass index; CI, confidence interval; ED, erectile dysfunction; OR, odds ratio; SD, standard deviation; WHOQOL, World Health Organization Quality of Life.

The final sample size for the regression was 300. There was a statistically significant positive association between age and ED (OR = 1.068; 95% CI = [1.035; 1.102]; *p* ≤ 0.001). The relative percentage of the WHOQOL psychological domain score had a statistically significant negative association with ED (OR = 0.645; 95% CI = [0.511; 0.814]; *p* ≤ 0.001). Regarding ethnicity, the reference category was white individuals. Compared with white participants, those who self‐identified as black had more than three times (OR = 3.119; 95% CI = [1.174; 8.283]; *p* = 0.022) the odds of experiencing ED. Those who self‐declared as belonging to other race/skin colors had greater odds of being in the ED group compared with those who self‐identified as white (OR = 2.942; 95% CI = [1.007; 8.591]; *p* = 0.048). The other variables or categories did not show statistically significant effects (Table 5). According to the $R_{\mathrm{MF}}^{2}$ value, the model explains 26.8% of the variation in the dependent variable (Table S1). The results suggest that, for the sample studied, individuals who self‐reported ethnicities other than white and older age had higher odds of having ED. A higher WHOQOL psychological score was associated with lower odds of having ED.

**TABLE 5:** Results from the binary logistic regression model considering the occurrence (no or yes) of erectile dysfunction (ED) as the dependent variable (*N* = 300).

Independent variables	Estimates	SE	*p*‐value	OR	95% CI
Intercept	‒0.004	1.953	0.998	0.996	[0.022; 45.799]
Age (years)	0.066	0.016	<0.001	1.068	[1.035; 1.102]
WHOQOL psychological (%)	‒0.439	0.119	<0.001	0.645	[0.511; 0.814]
BDI score	0.048	0.030	0.111	1.049	[0.989; 1.113]
BAI score	‒0.040	0.027	0.145	0.961	[0.911; 1.014]
Total testosterone (<300 to ≥300 ng/dL)	‒0.398	0.546	0.466	0.671	[0.230; 1.959]
AHI (≥15 to <15 events/h)	0.308	0.391	0.431	1.360	[0.633; 2.925]
Arterial hypertension (yes‒no)	‒0.331	0.429	0.441	0.718	[0.310; 1.665]
Diabetes (yes‒no)	0.700	0.500	0.162	2.015	[0.756; 5.372]
Ethnicity					
Black‒white	1.137	0.498	0.022	3.119	[1.174; 8.283]
Mixed race‒white	0.831	0.463	0.073	2.295	[0.927; 5.682]
Indigenous/Asian/others/unknown—white	1.079	0.547	0.048	2.942	[1.007; 8.591]
BMI					
Overweight–Normal	0.770	0.448	0.085	2.160	[0.898; 5.194]
Obese–Normal	0.545	0.516	0.291	1.725	[0.627; 4.744]

*Note*: ORs resulted from the comparison of having erectile dysfunction/not having erectile dysfunction.

Abbreviations: AHI, apnea‒hypopnea index; BAI, Beck Anxiety Index; BDI, Beck Depression Inventory; BMI, body mass index; CI, confidence interval; OR, odds ratio; SE, standard error; WHOQOL, World Health Organization Quality of Life.

#### 3.2.2 Mediation/moderation models

**FIGURE 4:**
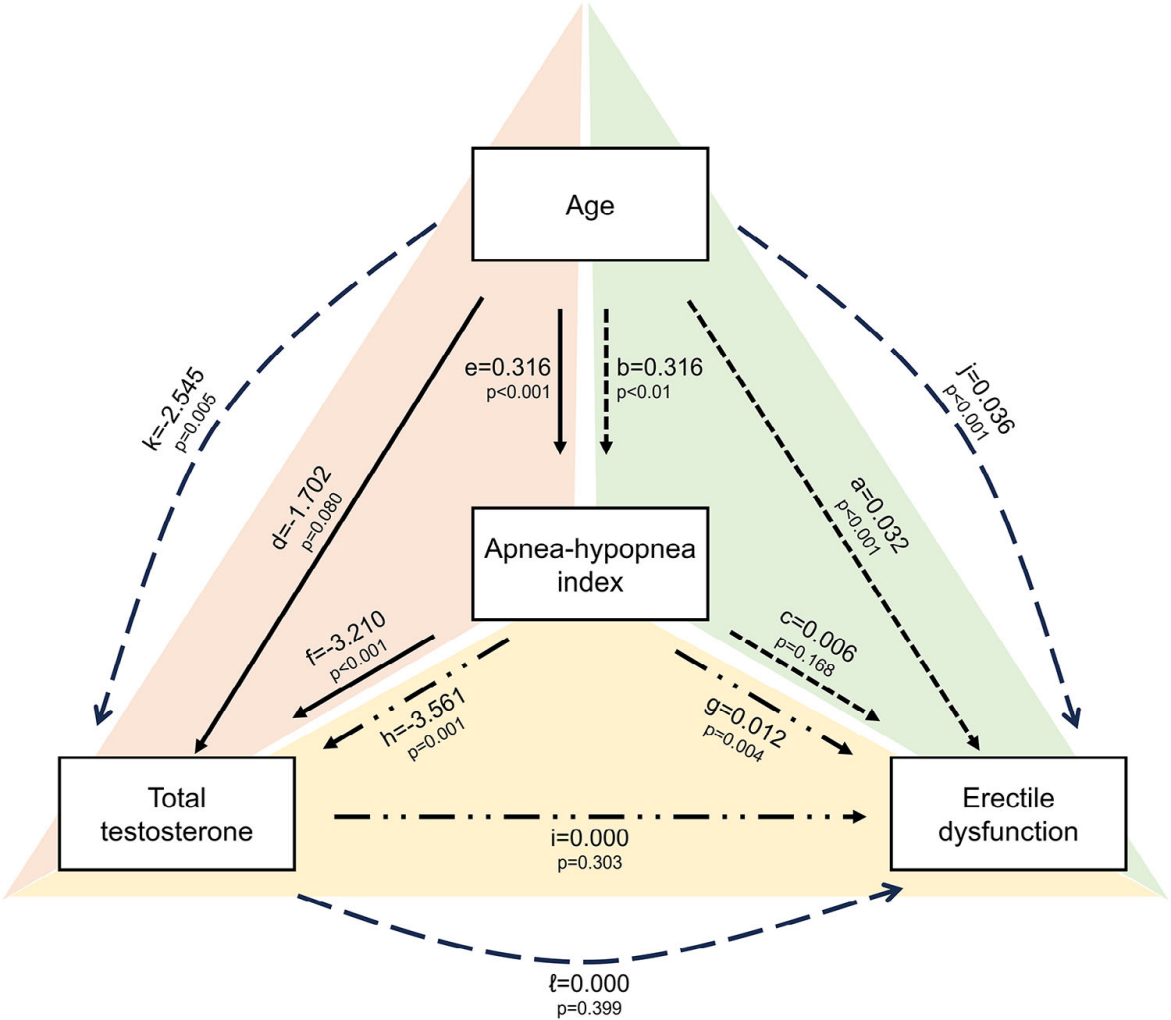
Summary of results from four mediation/moderation models. Models i, ii, and iii are represented in green, red, and yellow, respectively. Model iv includes the variables located at the vertices of the triangle. In models i, ii, and iv, only the direct effects were statistically significant (i. age → erectile dysfunction [ED], ii. apnea‒hypopnea index [AHI] → ED, and iv. age → ED), while in model ii, the effect of age on total testosterone was mediated through AHI.

Each of the four models developed evaluated the relationship between a trio of variables: age, AHI, total testosterone, and ED (Figure 4 and Table S2). In model i, the direct effect of age on ED was statistically significant (*a* = 0.032; *p* < 0.001), while the indirect effect through AHI was not significant (*b* × *c* = 0.002; *p* = 0.196). Model iii followed the same pattern, in which there was significance in the direct effect of AHI on ED (*g* = 0.012; *p* = 0.004) but not in the indirect effect through total testosterone (*h* × *i* = ‒0.001; *p* = 0.325). In model ii, the direct effect of age on total testosterone was not statistically significant (*d* = ‒1.702; *p* = 0.080). The effect of age on this hormone was mediated by AHI (*e* × *f* = ‒1.016; *p* = 0.002), resulting in a significant total effect (*d* + (*e* × *f*) = ‒2.718; *p* = 0.005). The model iv results were that age had a significant direct effect on ED (*j* = 0.036; *p* < 0.001), and the indirect effect did not reach statistical significance (*k* × *l* = ‒0.001; *p* = 0.419). Taken together, the mediation models suggest that the association between increased AHI and aggravated ED is not independent of age; the decrease in total testosterone levels because of age was not a direct effect but was mediated by AHI. Moreover, total testosterone was not a significant mediator of the effects of age and AHI on ED.

## 4 DISCUSSION

In the 8‐year follow‐up analysis, the overall incidence of ED of 10.55% in our sample, which was higher in men older than 50 years. The longitudinal model had age, depression symptoms, and low total testosterone concentration as significant predictors of higher odds of ED. The cross‐sectional analysis demonstrated that age, psychological quality of life, and race/skin color had statistically significant associations with increased odds of having ED.

A study with a cohort from Australia, consisting of men aged 35‒80 years, reported a 31.7% incidence rate of ED in a 5‐year follow‐up using the International Index of Erectile Function to diagnose dysfunction.^23^ The higher overall incidence demonstrated in this study may be because of the different target populations and their characteristics because of only participants aged ≥35 years were included.^23^ ED prevalences of 17.67% (95% CI = [13.6%; 22.2%]) and 20.06% (95% CI = [15.9%; 24.7%]) were identified in the 2015 and 2018 EPISONO surveys, respectively, and rates were higher in men older than 50 years old. A study involving men from several countries included in the multinational Men's Attitudes to Life Events and Sexuality study—Brazil, France, Italy, Mexico, Spain, the UK, Germany, and the United States—identified an overall prevalence of 16% with data from 2001, while in Brazil, it was 14% in the same study.^24^ The review by Kessler et al. found a global prevalence of 13.1%‒71.2% in studies carried out in Africa, Asia, North America, Oceania, and Europe^25^; however, no findings were described with respect to South America by the authors. Compared to the general prevalence of ED observed in our study with data from 2007 (17.08%),^6^ the prevalences of ED in 2015 and 2018 presented here remained similar when considering the confidence intervals, although an increasing trend can be noticed.

ED is a multifactorial condition, and its main endocrinal factor is related to low testosterone concentration, which negatively affects male sexual response.^26^ Testosterone contributes to heightened sexual desire, resulting in amplified frequencies of sexual activity. A higher testosterone concentration also increases occurrences of sleep‐related erections, and thus, concurrently participates in alterations to the structural composition of the corpora cavernosa.^26^ We observed an association between low total testosterone and ED in the longitudinal assessment, reinforcing the role of this hormone as a predictor for this sexual disorder.

Depression symptoms have been shown to be associated with ED, and we observed an association between these factors in the longitudinal analysis, but not in the cross‐sectional study. In a secondary analysis with data from the Diabetes Prevention Program (United States), individuals treated with metformin after 1‐year follow‐up presented an increase in total testosterone, which was associated with a decrease in BDI and BAI scores, that is, fewer depression and anxiety symptoms, respectively.^27^ In a study with a 3‐year follow‐up in a sample of men with diabetes, subjects with ED at baseline documented worse scores in quality of life (according to the SF‐36 scale), especially with respect to the physical components. The incidence of ED was related to a significant increase in depression symptoms.^28^ An EPISONO study, with cross‐sectional data from the baseline assessment (2007), revealed that individuals with depression symptoms and ED had lower and quality of life scores on the physiological domain, indicating the synergistic association of these conditions regarding quality of life.^29^ Latini et al. evaluated whether 1 year of ED therapy could improve quality of life, and the results confirmed this hypothesis, especially in the domains relating to the psychological impact of ED.^30^ Our results are aligned with previous studies that highlighted the link between ED and depression. More than 80 genes are common to both conditions, including some that participate in the morphological development of blood vessels, and others that are expressed in both the brain and corpus cavernosum tissues.^31^ A higher risk for ED was found in men exposed to depressive disorder.^32^, ^33^ Additionally, emotional and cognitive factors, which both influence and are impacted by quality of life, play a significant role in the development and management of ED.^34^ Individuals who present depression symptoms and worsened psychological well‐being have difficulty expressing and feeling the sensation of pleasure and desire, whether from an emotional or sexual point of view,^35^ which ultimately hinders sexual function and the occurrence of the erection itself, with a subsequent impact on quality of life. Considering the evidence from the literature together with our results, we can argue that the psychological domain of quality of life can have a bidirectional relationship with ED.

Data from the EPISONO study conducted in 2007 depicted that OSA was associated with ED, and that an AHI ≥15, a parameter that suggests moderate or severe OSA, also elevated the risk of having ED.^6^ In a study by Zhang et al.,^36^ male subjects with an AHI ≥30 showed poorer erectile function than those with an AHI <5. Total testosterone concentration was lower in those with an AHI ≥30.^36^ Contrary to our hypothesis, we observed a lack of association between AHI and ED, which may be attributed to several factors. First, AHI may not be the most appropriate metric for assessing OSA in this context, as it does not fully capture the complexity of disordered breathing during sleep. Second, the heterogeneity of OSA phenotypes suggests that only specific subgroups may be more susceptible to ED, a distinction that current classification methods may not yet be able to detect. Last, intermediary mechanisms such as vascular alterations, inflammatory responses, cardiac or metabolic comorbidities,^37^ and diabetes,^38^ may serve as a more direct link between OSA and ED rather than AHI itself. Nevertheless, the mediation/moderation models confirmed the effect of this sleep‐breathing parameter on ED, even though it was not independent of age. Our study assessed testosterone, depression symptoms, diabetes, quality of life, AHI, and aging. All these factors are, in some way, affected by the aging process. As expected, both the longitudinal and cross‐sectional analyses of our study indicated aging as a major risk for ED. The analysis revealed distinct longitudinal impacts of low testosterone concentration and aging, implying that age‐associated contributions to ED involve biological processes beyond those evaluated here. These could encompass unexamined cardiovascular, metabolic, or hormonal axis interactions.^37^, ^38^, ^39^

When comparing the findings of Andersen et al.^6^ with those of the present study, several important nuances emerge. In the 2007 EPISONO study, 17.08% of men reported complaints of ED. Interestingly, in the follow‐up conducted in 2015, we observed a similar prevalence rate of 10.55%. In the 2018 edition, an increase in ED prevalence was observed, reaching 20.05%. In all editions of the study, the highest incidence was consistently detected among men over 50 years of age, reinforcing the role of aging as a relevant factor in male sexual function. A particularly noteworthy point concerns the association between ED, OSA, and testosterone levels. In 2007, it was found that men with reduced testosterone or OSA presented a significantly increased risk of reporting ED, approximately fourfold in the former case and 2.1‐fold in the latter. However, at that time, the analysis did not allow for a clear determination of which variable played a predominant causal role. In the more recent 2018 study, the findings allowed us to infer that there is indeed a direct risk of ED development because of age, and that the reduction in total testosterone because of age was mediated by AHI. These results support the hypothesis that OSA may impair sexual function through hormonal pathways, particularly via androgen deficiency. Although the studies differ in methodology and timing, their findings are complementary and point in the same direction, namely, the need to consider hormonal and sleep‐related respiratory factors in an integrated manner when investigating the etiology of ED, especially in older men.

Previous research indicated that continuous positive airway pressure (CPAP) therapy ameliorates ED symptoms in patients with both OSA and ED.^40^, ^41^ A study highlighted a more substantial therapeutic effect of CPAP in individuals concurrently experiencing depression or anxiety.^42^ Beyond its impact on ED, CPAP has demonstrated efficacy in alleviating depressive and anxiety symptoms.^43^, ^44^ However, the mechanism underlying CPAP's influence on ED is not fully understood; it is uncertain whether this effect arises directly or is secondary to enhancements in mental health or quality of life.

Limitations of the present study include the reliance on self‐report to determine the presence of ED without urological assessment, which can incur in underestimation of ED, and the inability to discern the etiology of ED. However, this method of assessing ED has demonstrated clinical appropriateness for decades,^12^ and it is a valid indirect indicator of this sexual dysfunction. The questionnaire data were meticulously analyzed in conjunction with a comprehensive full‐night PSG, the gold standard method for sleep evaluation. The analysis incorporated cross‐sectional and longitudinal data obtained from the EPISONO study, including BDI scores, the individual's comorbidity history, and an evaluation of testosterone. Future interventional studies should be conducted to better understand the role of depressive symptoms, quality of life, and diabetes in relation to ED, with a focus on interventional studies assessing the impact of CPAP therapy across different age groups. This may provide a deeper understanding of the interplay between AHI, testosterone levels, and ED.

## 5 CONCLUSIONS

Between 2007 and 2015, aging, low total testosterone, psychological quality of life, and symptoms of depression were associated with the presence of erectile dysfunction. This 8‐year follow‐up found a general incidence of erectile dysfunction of 10.55% in a representative sample from São Paulo city. Compared to 2007, the general prevalence of erectile dysfunction in 2018 with a new sample was slightly higher (20.06%). The cross‐sectional analysis highlights the importance of aging, psychological quality of life, and race/skin color when considering the prevalence of erectile dysfunction. Mediation models indicated that increasing apnea‒hypopnea index had an association with diminished total testosterone, and a significative association between apnea‒hypopnea index and erectile dysfunction that was not independent of age. These findings underscore the need for healthcare providers to adopt a comprehensive approach to erectile dysfunction management, integrating both physical and psychological aspects, as well as sleep‐related factors, to ensure more effective diagnosis and treatment.

## AUTHOR CONTRIBUTIONS

*Conceptualization, methodology, validation, investigation, resources, writing, writing—review and editing, project administration, and supervision*: Monica Levy Andersen. *Methodology, validation, investigation, data curation, formal statistical analysis, writing—original draft, and writing—review and editing*: Allan Saj Porcacchia. *Methodology, validation, investigation, data curation, formal statistical analysis, writing—original draft, writing—review and editing, and visualization*: Guilherme Luiz Fernandes. *Writing—original draft and writing—review and editing*: Tathiana A. Alvarenga. *Conceptualization, project administration, resources, writing—review and editing, and supervision*: Sergio Tufik.

## CONFLICT OF INTEREST STATEMENT

The authors declare no conflicts of interest.

## Supporting information



Supporting Information

Supporting Information

## Data Availability

The data that support the findings of this study are available upon request from the corresponding author, M.L.A. The data are not publicly available because they contain information that could compromise the privacy of research participants.
