# Supplementary material for: Unraveling the intersection of sleep disorders and erectile dysfunction: Outcomes from two EPISONO editions
Source: Andrology. 2025 Jun 4;14(2):385–97. doi: 10.1111/andr.70067 (PMC12842842; doi:10.1111/andr.70067)
Supplement: Supplementary file 1 — Supporting Information [file ANDR-14-385-s001.docx]

Supplementary Table 1. Parameters of the statistical models presented in the article.

| **Binary logistic regression model parameters** | | | |
| --- | --- | --- | --- |
|  | VIF | Tolerance | R²_MF_ |
| *Model* |  |  | 0.268 |
| Age | 1.219 | 0.821 |  |
| WHOQOL psychological | 1.197 | 0.835 |  |
| BDI score | 1.390 | 0.719 |  |
| BAI score | 1.244 | 0.804 |  |
| Total testosterone | 1.055 | 0.948 |  |
| AHI | 1.079 | 0.927 |  |
| Arterial hypertension | 1.149 | 0.870 |  |
| Diabetes | 1.092 | 0.916 |  |
| Ethnicity | 1.026 | 0.975 |  |
| BMI | 1.075 | 0.930 |  |
| Abbreviations: AHI=apnea-hypopnea index; BAI=Beck Anxiety Inventory; BDI=Beck Depression Inventory; BMI=body mass index; CI=confidence interval; OR=odds ratio; SE=standard error; WHOQOL=The World Health Organization Quality of Life; VIF=variance inflation factor; R²_MF_=McFadden's R². | | | |
