# Supplementary material for: Unraveling the intersection of sleep disorders and erectile dysfunction: Outcomes from two EPISONO editions
Source: Andrology. 2025 Jun 4;14(2):385–97. doi: 10.1111/andr.70067 (PMC12842842; doi:10.1111/andr.70067)
Supplement: Supplementary file 2 — Supporting Information [file ANDR-14-385-s002.docx]

Supplementary Table S2. Mediation/moderation model results.

|  | | | |  |
| --- | --- | --- | --- | --- |
| Effect | Estimate | Std. Error | p-value |  |
| *Model i* | | | |  |
| Age → ED (a) | 0.032 | 0.006 | **<0.001** |  |
| Age → AHI (b) | 0.316 | 0.083 | **<0.001** |  |
| AHI → ED (c ) | 0.006 | 0.005 | 0.168 |  |
| b*c | 0.002 | 0.002 | 0.196 |  |
| Total effects | 0.034 | 0.006 | **<0.001** |  |
| *Model ii* | | | |  |
| Age → Total testosterone (d) | -1.702 | 0.971 | 0.08 |  |
| Age → AHI (e) | 0.316 | 0.069 | **<0.001** |  |
| AHI → Total testosterone (f) | -3.21 | 0.774 | **<0.001** |  |
| e*f | -1.016 | 0.329 | **0.002** |  |
| Total effects | -2.718 | 0.965 | **0.005** |  |
| *Model iii* | | | |  |
| AHI → ED (g) | 0.012 | 0.004 | **0.004** |  |
| AHI → Total testosterone (h) | -3.561 | 1.091 | **0.001** |  |
| Total testosterone → ED (i) | 0 | 0 | 0.303 |  |
| h*i | -0.001 | 0.001 | 0.325 |  |
| Total effects | 0.011 | 0.004 | **0.007** |  |
| *Model iv* | | | |  |
| Age → ED (j) | 0.036 | 0.006 | **<0.001** |  |
| Age → Total testosterone (k) | -2.545 | 0.907 | **0.005** |  |
| Total testosterone → ED (l) | 0 | 0 | 0.399 |  |
| k*l | -0.001 | 0.001 | 0.419 |  |
| Total effects | 0.035 | 0.006 | **<0.001** |  |
| AHI=Apnea-hypopnea index; ED=Erectile dysfunction. Bold values indicate statistically significant effects at the p<0.05 level. | | | |  |
|  |  |  |  |  |
